# Intricate crosstalk between MYC and non‐coding RNAs regulates hallmarks of cancer

**DOI:** 10.1002/1878-0261.12409

**Published:** 2018-12-05

**Authors:** Lotteke J. Y. M. Swier, Agnieszka Dzikiewicz‐Krawczyk, Melanie Winkle, Anke van den Berg, Joost Kluiver

**Affiliations:** ^1^ Department of Pathology and Medical Biology University of Groningen University Medical Center Groningen The Netherlands; ^2^ Institute of Human Genetics Polish Academy of Sciences Poznan Poland

**Keywords:** lncRNA, miRNA, MYC, ncRNA

## Abstract

Myelocytomatosis viral oncogene homolog (MYC) plays an important role in the regulation of many cellular processes, and its expression is tightly regulated at the level of transcription, translation, protein stability, and activity. Despite this tight regulation, MYC is overexpressed in many cancers and contributes to multiple hallmarks of cancer. In recent years, it has become clear that noncoding RNAs add a crucial additional layer to the regulation of MYC and its downstream effects. So far, twenty‐five microRNAs and eighteen long noncoding RNAs that regulate MYC have been identified. Thirty‐three miRNAs and nineteen lncRNAs are downstream effectors of MYC that contribute to the broad oncogenic role of MYC, including its effects on diverse hallmarks of cancer. In this review, we give an overview of this extensive, multilayered noncoding RNA network that exists around MYC. Current data clearly show explicit roles of crosstalk between MYC and ncRNAs to allow tumorigenesis.

**Abbreviations**
AGO2: Argonaute 2
Akt: AKT serine/threonine kinase
AMBRA1: activating molecule in Beclin‐1‐regulated autophagy
AMPK: AMP‐activated kinase
ARE: A+U‐rich element
ATP: adenosine triphosphate
AUF1: A + U‐rich element RNA‐binding protein
BAD: BCL2‐associated agonist of cell death
BCYRN1: brain cytoplasmic RNA 1
CASC11: cancer susceptibility candidate 11
CCAT: colon cancer‐associated transcript
CCN: cyclin
CDC: cell division cycle
CDK: cyclin‐dependent kinase
CDKN: cyclin‐dependent kinase inhibitor
ceRNA: competing endogenous RNA
CIP2A: cancerous inhibitor of protein phosphatase 2A
CNBP: cellular nucleic acid‐binding protein
CONCR: cohesion regulator noncoding RNA
CTCF: CCCTC‐binding factor
CTGF: connective tissue growth factor
DANCR: differentiation antagonizing non‐protein‐coding RNA
DDX11: DEAD/H‐box helicase 11
DNMT3a: DNA methyltransferase 3a
EMT: epithelial–mesenchymal transition
EPIC1: epigenetically induced lncRNA 1
EZH2: enhancer of zeste 2 polycomb repressive complex 2
Fbxw7: F‐box and WD repeat‐containing protein 7
FILNC1: FoxO‐induced lncRNA 1
G6PD: glucose‐6‐phosphate dehydrogenase
GADD45A: growth arrest and DNA damage‐inducible alpha
GHET1: gastric carcinoma high expressed transcript 1
GIT2: G‐protein‐coupled receptor kinase interactor 2
GLS: glutaminase
GLUT: glucose transporter member
GSK3β: glycogen synthase kinase 3 beta
HDAC3: histone deacetylase 3
HIF1α: hypoxia‐inducible factor 1α
HK2: hexokinase 2
HMGA2: high‐mobility group AT‐hook 2
hnRNP: heterogeneous nuclear ribonucleoprotein
HOTAIR: homeobox transcript antisense intergenic RNA
HuR: RNA‐binding protein human antigen R
IDH1: isocitrate dehydrogenase 1
IGF2BP: insulin‐like growth factor 2 mRNA‐binding protein
IL‐6: interleukin 6
IRES: internal ribosome entry segment
JAK: Janus kinase
LAST: lncRNA‐assisted stabilization of transcripts
LDHA: lactate dehydrogenase A
LIFR: leukemia inhibitory factor receptor
Lin28B: Lin28 homolog B
Linc‐RoR: lincRNA regulator of reprogramming
lncRNA: long noncoding RNA
MAX: MYC‐associated protein X
MIF: MYC inhibitory factor
MINCR: MYC‐induced long noncoding RNA
miRNA: microRNA
MMP: metalloproteinase
mTOR: mechanistic target of rapamycin kinase
MYCBP: MYC‐binding protein
MYC: myelocytomatosis viral oncogene homolog
MYU: MYC‐induced lncRNA
ncRNA: noncoding RNA
NEAT1: nuclear‐enriched abundant transcript 1
NFκB: nuclear factor kappa B
OGT: *O*‐GlcNAc transferase
PCAT‐1: prostate cancer‐associated ncRNA transcript 1
PCGEM1: prostate cancer gene expression marker 1
PDH: pyruvate dehydrogenase
PDIA3P: protein disulfide isomerase family A member 3 pseudogene 1
PDK: pyruvate dehydrogenase kinase
PFK: phosphofructokinase
PI3K: phosphatidylinositol‐4,5‐biphosphate 3 kinase
PKM2: pyruvate kinase M2
PP2A: protein phosphatase 2A
PPP: pentose phosphate pathway
PTEN: phosphatase and tensin homolog
Puma: p53 upregulated modulator of apoptosis
PVT1: plasmacytoma variant translocation 1
RB1: RB transcriptional corepressor 1
RISC: RNA‐induced silencing complex
ROCK: Rho‐associated coiled‐coil‐containing protein kinase
SMAD: mothers against decapentaplegic homolog
SNAI: snail family transcriptional repressor 1/2
SNHG: small nucleolar RNA host gene
SOCS5: suppressor of cytokine signaling 5
SPOP: speckle‐type POZ
STAT3: signal transducer and activator of transcription 3
SUFU: suppressor of fused homolog
TCA: tricarboxylic acid cycle
TCF7L2: transcription factor 7 like 2
TFAP4: transcription factor AP‐4
TFDP2: transcription factor Dp‐2
TGFBR2: TGF‐β receptor type II
TGF‐β: transforming growth factor‐β
THBS: thrombospondin‐1
THOR: testis‐associated highly conserved oncogenic long noncoding RNA
TOB2: transducer of ERBBS
TUSC2: tumor suppressor candidate 2
UBE3C: ubiquitin protein ligase E3C
USP28: ubiquitin‐specific peptidase 28
VASH2: vasohibin‐2
VEGF: vascular endothelial growth factor
Wif1: Wnt inhibitory factor 1
YAP: yes‐associated protein
ZEB: zinc finger E‐box‐binding homeobox
ZNF281: zinc finger protein 281

## 1 Introduction

The *MYC* gene family consist of three members, that is, c‐MYC, n‐MYC, and l‐MYC. c‐MYC forms a central hub in all cells by regulating many cellular processes, while n‐MYC and l‐MYC are more tissue‐specific regulators. MYC proteins are overexpressed in more than half of all human cancers, including lung, breast, and colon cancers (Albihn *et al*., 2010). This overexpression is caused by diverse mechanisms including amplifications, translocations, and epigenetic alterations (Kalkat *et al*., 2017). In this review, we will focus on c‐MYC, hereafter referred to as MYC.

MYC belongs to the basic helix–loop–helix superfamily and functions as a transcription factor. Upon dimerization with its binding partner MAX, the MYC‐MAX dimer binds to E‐box sequences in the promoter region of its targets genes, thereby activating transcription of these genes (Tu *et al*., 2015). In addition to interacting with MAX, MYC can also interact with other transcription factors, histone‐modifying enzymes, and DNA methyltransferases to repress transcription. MYC regulates the transcription of many different genes, which include protein‐coding as well as noncoding genes (Dang, 2012; Hart *et al*., 2014; Winkle *et al*., 2015). These noncoding genes can include various RNA molecules, for example, miRNAs and lncRNAs.

miRNAs are noncoding, regulatory RNA molecules of about 22 nucleotides in length. A miRNA is transcribed as a longer primary transcript, which is processed in two steps into a mature single‐stranded miRNA and subsequently incorporated into the RISC. The miRNA guides the RISC complex to its target mRNA by recognition of a complementary sequence, most often in the 3′ UTR. Usually, conserved Watson–Crick pairing with nucleotides 2–7 of the miRNA, the so‐called seed region, is essential for target recognition (Bartel, 2009). Binding to the target mRNA will subsequently result in mRNA cleavage by AGO2 in case the miRNA has high complementarity with the binding site region on the mRNA. In case of a low level of complementarity, binding will lead to translational repression.

LncRNAs are defined as noncoding RNA molecules of more than 200 nucleotides in length. Their expression is often tissue specific or cell type specific, and their transcripts can have subcellular compartment‐specific localizations. Together, this restricts their function to specific cell types and locations. LncRNAs can regulate gene expression at the transcriptional and post‐transcriptional level, as well as by modulating protein stability, localization, and functionality via diverse mechanisms. In the nucleus, lncRNAs can regulate transcription of nearby genes in *cis* or of more distant genes in *trans*, for example, by recruiting transcription factors, chromatin‐modifying complexes, or heterogeneous nuclear ribonucleoprotein (hnRNP) complexes. LncRNAs residing in the cytoplasm can modulate mRNA stability, translation efficiency, or protein stability, localization, or activity. Cytoplasmic lncRNAs can act as decoys to sequester RNA binding proteins or miRNAs (sponges or ceRNAs) or interfere with post‐translational modifier proteins (Chen, 2016; Schmitt and Chang, 2016).

Over the last decades, it has become clear that MYC is not only regulated by and regulates many protein‐coding genes, but this extensive network also includes the family of ncRNAs. The overall aim of this review was to present an overview of the intricate crosstalk between ncRNAs and MYC. We first focus on ncRNAs acting upstream of MYC by regulating its transcription, translation, and activity. In addition, we focus on ncRNAs acting downstream of MYC and pinpoint their contributions to crucial hallmarks of cancer.

## 2 ncRNAs regulating MYC

### 2.1 miRNAs regulating MYC

**Figure 1:**
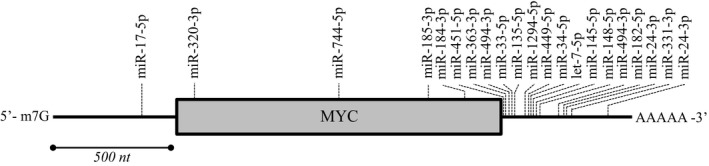
miRNA‐binding sites on the MYC mRNA. Schematic representation of the MYC mRNA with the binding sites of the MYC‐regulating miRNAs indicated. Only miRNAs for which binding to the mRNA was proven at least by reporter assay have been implemented in the figure. The miRNAs let‐7a/b/c/e/f‐5p and miR‐98‐5p of the let‐7 family (Bueno *et al*., 2011; Kim *et al*., 2009), miR‐24‐3p (Lal *et al*., 2009), miR‐33b‐5p (Takwi *et al*., 2012), miR‐34a/c‐5p (Christoffersen *et al*., 2010; Kong *et al*., 2008), miR‐145‐5p (Sachdeva *et al*., 2009), miR‐135b‐5p (Liu *et al*., 2014), miR‐148a‐5p (Han *et al*., 2013), miR‐182a‐5p (Huang *et al*., 2017), miR‐331‐3p (Bueno *et al*., 2011), miR‐363‐3p (Bueno *et al*., 2011), miR‐449c‐5p (Miao *et al*., 2013), miR‐451‐5p (Li *et al*., 2011), miR‐494‐3p (Zhang *et al*., 2012b), and miR‐1294‐5p (Liu *et al*., 2015a) target the MYC mRNA by binding to its 3′ UTR, while miR‐17‐5p binds to the 5′ UTR (Liu *et al*., 2016) and miR‐184‐3p (Zhen *et al*., 2013), miR‐185‐3p (Liao and Liu, 2011), miR‐320b‐3p (Wang *et al*., 2015), and miR‐744‐5p (Lin *et al*., 2014) bind to the MYC ORF.

In total, twenty‐five miRNAs belonging to twenty different seed families have been described to directly regulate MYC (Fig. 1). Most of the miRNAs bind to the MYC transcript in a canonical fashion, that is, with so‐called seed‐containing binding sites in the 3′UTR. Binding of let‐7b/c‐5p is enhanced by adjacent binding of the RNA‐binding protein HuR, which makes the miRNA binding site accessible (Kim *et al*., 2009). One of the two miR‐24‐3p binding sites is seed‐containing, while the other less‐efficient site is ‘seedless’ and has extensive complementarity at the 3′‐end of the miRNA (Lal *et al*., 2009). MiR‐17‐5p was shown to bind to the 5′ UTR of the MYC mRNA (Liu *et al*., 2016), while miR‐184‐3p (Zhen *et al*., 2013), miR‐185‐3p (Liao and Liu, 2011), miR‐320b‐3p (Wang *et al*., 2015), and miR‐744‐5p (Lin *et al*., 2014) bind to the MYC ORF.

Next to regulating MYC in a direct fashion, miR‐24‐3p can also influence MYC protein levels indirectly by targeting OGT. OGT can *O*‐GlcNAcylate the MYC protein and thereby increase its stability (Liu *et al*., 2017). A second miRNA that can act indirectly on MYC is miR‐375‐3p, which targets CIP2A. CIP2A prevents phosphorylation of Ser62 on MYC by PP2A and thereby prevents degradation of MYC (Jung *et al*., 2013). So, miR‐24‐3p and miR‐375‐3p can downregulate MYC protein levels indirectly by targeting OGT and CIP2A, respectively.

Many of the miRNAs that can directly downregulate MYC by binding to the MYC mRNA, show reduced levels in cancer. The decreased expression of these miRNAs can thus contribute to the high levels of MYC as commonly observed in cancer. Examples are the let‐7‐5p family, miR‐148a‐5p, miR‐331‐3p, and miR‐363‐3p, which are downregulated in Burkitt lymphoma compared to normal lymph nodes (Bueno *et al*., 2011). A well‐known exception is miR‐17‐5p, which is part of the oncogenic miR‐17~92 cluster that is often upregulated in MYC‐driven cancers. As too high MYC levels are potentially dangerous for cancer cells, targeting of MYC by miR‐17‐5p may be a means to maintain optimal MYC levels and sustain continuous tumor growth (Liu *et al*., 2016).

### 2.2 lncRNAs regulating MYC

**Figure 2:**
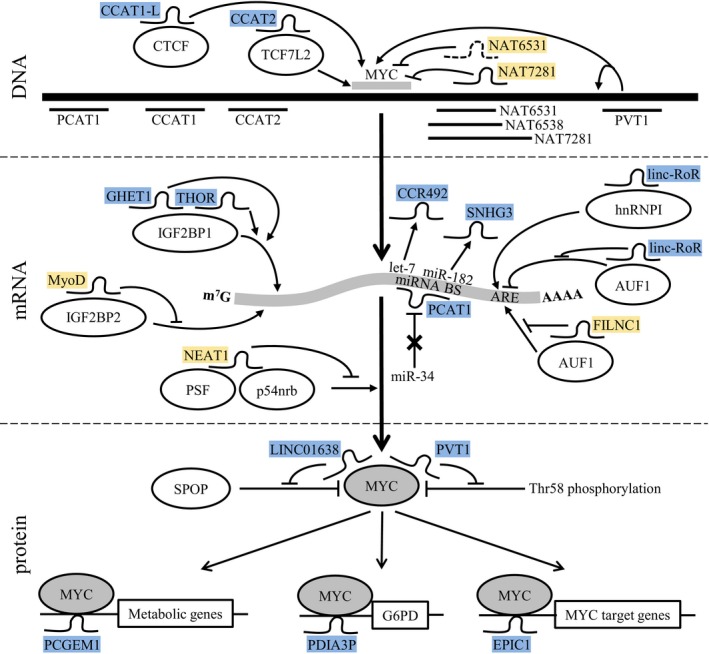
lncRNAs regulating MYC at the DNA, mRNA, or protein levels. LncRNAs and their interaction partners involved in regulation of MYC transcription, translation, stability, and functionality at protein level are indicated. The genomic region around MYC and the MYC mRNA (central thick and curved gray line) are not drawn to scale. LncRNAs are indicated by curved lines and proteins by ellipses. LncRNAs highlighted in blue indicate that they stimulate and lncRNA highlighted in yellow indicate that they repress MYC transcription, translation, stability, or functionality, and the arrows indicate stimulating or repressing effects on MYC.

Expression of MYC is controlled at the level of transcription, translation, and protein stability. Several lncRNAs have been demonstrated to play a role in these regulatory processes. Here, we describe the lncRNAs with a well‐characterized role in MYC regulation (Fig. 2).

#### 2.2.1 LncRNAs regulating MYC transcription in cis

Besides the MYC gene, the 8q24 region harbors several noncoding genes that can regulate MYC transcription. CCAT1‐L transcript variant of the CCAT1 gene and CCAT2 are specifically expressed in colorectal cancer (Ling *et al*., 2013; Xiang *et al*., 2014). CCAT1‐L is a nuclear lncRNA that accumulates in distinct nuclear foci near its site of transcription. Knockdown of CCAT1‐L reduced, while overexpression enhanced transcription of MYC in *cis*. This regulatory effect on MYC was attributed to the spatial proximity of the CCAT1‐L locus with the MYC promoter. Indeed, reduced chromatin loop formation between the CCAT1‐L and MYC loci was observed upon knockdown of CCAT1‐L transcription. The loop formation was dependent on interaction of CCAT1‐L with CTCF, which enhanced binding of CTCF to the MYC locus (Xiang *et al*., 2014). CCAT2 regulates MYC by enhancing the activity of TCF7L2, a transcription factor for MYC (Ling *et al*., 2013). Thus, both CCAT1‐L and CCAT2 positively regulate MYC transcription.

Interaction between an enhancer region downstream the first transcriptional start site of PVT1 and the PVT1 promoter itself has tumor suppressor activity by reducing MYC transcription (Cho *et al*., 2018). Silencing of the PVT1 promoter increased MYC expression independent of the PVT1 transcript itself. The underlying mechanism has been identified as a competition between the PVT1 promoter and the MYC promoter for interaction with the intragenic enhancer region in the PVT1 locus. Under normal conditions, these enhancers preferentially bind to the PVT1 promoter. Silencing of the PVT1 promoter allowed interaction of enhancers with the MYC promoter, leading to increased MYC transcription. Importantly, this effect is restricted to cells where MYC forms chromatin loops with PVT1, for example, breast cancer, as opposed to colorectal cancer or cervical carcinoma cells where MYC loops to the CCAT1 enhancer.

The levels of three partially overlapping lncRNA transcripts antisense to the 3′ distal region of MYC, NAT6531, NAT6538, and NAT7281, are regulated by histone H3 acetylation in prostate cancer cells. Under normal conditions, NAT6531 is expressed and processed by DICER into several short RNAs, which have a repressive effect on MYC transcription, possibly by binding to the MYC promoter and intron 1 through partial sequence complementarity. Partial inhibition of histone deacetylation shifts transcription from NAT6531 to NAT6538, and this releases the block on MYC transcription. Strong inhibition of histone deacetylation results in transcription of the longer NAT7281, which strongly represses MYC transcription (Napoli *et al*., 2017).

#### 2.2.2 LncRNAs controlling MYC mRNA stability and translation

IGF2BPs enhance mRNA stability and promote translation by binding to the MYC mRNA (Huang *et al*., 2018). A number of cell type‐specific lncRNAs have been identified that modulate this interaction. Interaction of IGF2BP1 with lncRNA GHET1 in gastric cancer and THOR in renal and skin cancer increased MYC mRNA and protein levels (Liu *et al*., 2018; Yang *et al*., 2014; Ye *et al*., 2018). In contrast, binding of the skeletal muscle‐specific lncRNA lncMyoD to IGF2BP2 decreased MYC mRNA levels by preventing binding of IGF2BP2 to MYC mRNA (Gong *et al*., 2015).

Binding of AUF1 to an ARE site in the 3′UTR of the MYC transcript can both positively and negatively affect MYC levels, depending on the cell‐type. In normal kidney cells, FILNC1 acts as a decoy for AUF1 preventing binding of AUF1 to the MYC mRNA, thereby resulting in low MYC protein levels. In renal cancer, FILNC1 is downregulated, resulting in an AUF1‐dependent increase in MYC protein levels (Xiao *et al*., 2017). In breast and colon cancers, binding of linc‐RoR to AUF1 inhibits binding of AUF1 to MYC mRNA and thereby increases MYC levels (Huang *et al*., 2015). It is currently unclear why sequestering of AUF1 has opposite effects on MYC levels in these different cell types. In addition, linc‐RoR facilitates binding of RNA binding protein hnRNP‐I to MYC mRNA and this also enhances MYC protein levels.

MYC can be translated using an IRES in case the regular cap‐dependent translation is compromised. This requires binding of the IRES trans‐acting factors PSF and p54nrb (Cobbold *et al*., 2008). These factors are sequestered by lncRNA NEAT1 to the paraspeckles. In HeLa cells, depletion of NEAT1 during nucleolar stress released PSF and p54nrb from paraspeckles and allowed IRES‐dependent translation of MYC (Shen *et al*., 2017).

LncRNAs can also stimulate MYC mRNA translation by competing with MYC‐regulating miRNAs. This has been shown for PCAT‐1, which competes with miR‐34a‐5p for interaction with its binding site in the 3′ UTR of the MYC mRNA (Prensner *et al*., 2011, 2014). The effect of PCAT‐1 can be antagonized by miR‐3667‐3p, which targets PCAT‐1.

#### 2.2.3 LncRNAs affecting MYC protein stability and activity

The stability of MYC protein can be increased by two lncRNAs that both prevent its degradation, but via distinct mechanisms. In contrast to the tumor‐suppressive role of the PVT1 promoter, the PVT1 transcript can act as an oncogene. PVT1 stabilizes the MYC protein by preventing phosphorylation of threonine 58, which is a signal for its degradation (Tseng *et al*., 2014). LINC01638 prevents MYC protein degradation by preventing binding of E3 ubiquitin ligase adapter SPOP to MYC (Luo *et al*., 2018).

Three lncRNAs modulate interaction of MYC with (subsets of) its target genes by directly binding to MYC. PCGEM1 is a prostate‐specific lncRNA, which together with MYC co‐occupies the promoter regions of several metabolic genes documented to be MYC targets. Knockdown of PCGEM1 reduced recruitment of MYC to the promoters of these PCGEM1‐dependent metabolic genes without affecting MYC protein levels (Hung *et al*., 2014). Thus, PCGEM1 affects the metabolic state of cancer cells by enhancing MYC occupancy at the promoters of several metabolic genes. LncRNA PDIA3P regulates the metabolic state of multiple myeloma cells via induction of G6PD, an enzyme crucial for promoting the PPP flux (Yang *et al*., 2018). This effect is achieved by interaction of PDIA3P with MYC and promoting MYC binding to the G6PD promoter. Together with MYC, lncRNA EPIC1 co‐occupies the promoters of > 97% of EPIC1‐regulated genes involved in cell cycle progression, and thereby regulates transcriptional activity of these genes in breast cancer cells (Wang *et al*., 2018).

From the studies presented here, lncRNAs emerge as important regulators of MYC expression and activity, either directly or indirectly by interacting with proteins. Often, these lncRNAs are deregulated in cancer and promote high MYC levels and activity. Since expression of lncRNAs is highly cell type specific, many of the lncRNA‐MYC interactions are restricted to certain tissues. Future studies will likely broaden the repertoire of lncRNAs regulating MYC and improve the understanding of the underlying mechanisms in normal and cancer cells.

### 2.3 Feedback loops on MYC

Next to the more straightforward regulation of MYC by ncRNAs as described above, more complex feedback loops between MYC and MYC‐regulating ncRNAs have been identified. These include feedback loops that involve MYC‐regulated miRNAs, as well as MYC‐regulated lncRNAs that act as sponges for MYC‐regulating miRNAs.

#### 2.3.1 Feedback loops involving MYC‐regulated miRNAs

Several miRNAs that regulate MYC can be induced or repressed by MYC as well, resulting in the formation of feedback loops. Examples of this are the feedback loops between MYC and MYC‐induced miR‐7‐5p (Capizzi *et al*., 2017; Chou *et al*., 2010), miR‐17‐5p (Liu *et al*., 2016), and miR‐185‐3p (Liao and Liu, 2011). For miR‐7‐5p, a positive feedback loop is formed via the miR‐7‐5p target AMBRA1, which promotes dephosphorylation of Ser62 on MYC upon binding to PP2A. This leads to stimulation of proteosomal degradation of MYC (Capizzi *et al*., 2017; Cianfanelli *et al*., 2015). In this way, miR‐7‐5p indirectly enhances MYC protein stability and promotes its own MYC‐mediated transcription. MiR‐17‐5p and miR‐185‐3p were shown to directly target MYC mRNA resulting in a negative feedback loop (Liao and Liu, 2011; Liu *et al*., 2016).

Positive feedback loops that result in sustaining high MYC expression also involve MYC‐repressed miRNAs. Let‐7a‐5p, miR‐34a‐5p, miR‐148a‐5p, miR‐363‐3p, and miR‐451‐5p are examples of MYC‐repressed miRNAs that can directly repress MYC translation (Bommer *et al*., 2007; Bueno *et al*., 2011; Christoffersen *et al*., 2010; Ding *et al*., 2018; Han *et al*., 2013; Sampson *et al*., 2007). Besides MYC, miR‐363‐3p also targets USP28, a de‐ubiquitinase involved in MYC stabilization (Han *et al*., 2013). MiR‐22 forms a feedback loop with MYC by targeting the MYCBP transcript, which encodes a positive regulator of MYC transcriptional activity (Xiong *et al*., 2010). In hepatocellular carcinoma, repression of liver‐specific miR‐122‐5p results in derepression of the miR‐122 targets E2F1 and its interaction partner TFDP2 (Wang *et al*., 2014). Both targets are involved in the induction of MYC transcription, creating another feedback loop. MiR‐200b‐3p participates in a feedback loop that involves MYC protein stability by targeting Akt2 mRNA (Lv *et al*., 2017). Akt2 represses the activity of GSK3β, an enzyme that destabilizes the MYC protein by phosphorylation of threonine residue 58. Thus, by repressing miR‐200b‐3p, MYC ensures inhibition of GSK3β, thereby stimulating its own stability. In contrast, MYC‐repressed miR‐30a‐5p is involved in a negative feedback loop by targeting UBE3C mRNA, a protein that can ubiquitinate MYC for proteosomal degradation (Chang *et al*., 2008; Xiong *et al*., 2016).

#### 2.3.2 Feedback loops involving MYC‐regulated lncRNAs acting as miRNA sponges

The functions of several MYC‐regulating miRNAs can be antagonized by MYC‐regulated lncRNAs, which act as sponges. By sequestering those miRNAs, the following MYC‐induced lncRNAs ensure high MYC levels and create a positive feedback loop on MYC: CCAT1‐S, the short isoform of CCAT1‐L (let‐7a/b/c/e‐5p) (Deng *et al*., 2015), DANCR (miR‐33b‐5p) (Ma *et al*., 2018), H19 (let‐7a/b‐5p) (Peng *et al*., 2017; Zhou *et al*., 2017), linc00176 (miR‐185‐5p) (Tran *et al*., 2017), and SNHG3 (miR‐182a‐5p) (Huang *et al*., 2017). Another lncRNA that ensures high MYC levels by sequestering miRNAs of the let‐7‐5p family without being regulated by MYC is lincRNA CCR492 (Maldotti *et al*., 2016). In contrast, the MYC‐induced lncRNA‐MIF reduces MYC levels and creates a negative feedback loop by sequestering miR‐586 (Zhang *et al*., 2016a). This miRNA targets the mRNA encoding E3 ubiquitin ligase Fbxw7, which stimulates MYC degradation. Although this does not seem beneficial for cancer cells, it might be that with the overall broad effects of MYC, lncRNA‐MIF is an additional factor in fine‐tuning the most optimal MYC levels.

## 3 MYC‐regulated ncRNAs involved in five important hallmarks of cancer

The C13ORF25 RNA also known as the primary transcript of the oncogenic miR‐17~92 cluster was identified as being MYC‐induced in 2005 (He *et al*., 2005; O'Donnell *et al*., 2005). The induction of this cluster is achieved by binding of MYC together with E2F1‐3 transcription factors to its promoter (Sylvestre *et al*., 2007; Woods *et al*., 2007). The miR‐17~92 cluster has two paralogs: the miR‐106a~363 cluster and the miR‐106b~25 cluster (Tanzer and Stadler, 2004). The miR‐106b~25 cluster is also regulated by E2F1 in combination with MYC (Petrocca *et al*., 2008). In 2008, multiple MYC‐repressed miRNAs were identified using a human and a mouse B‐cell lymphoma model (Chang *et al*., 2008). MYC represses expression of specific pri‐miRNAs by binding to their promoter regions and recruitment of HDAC3 (miR‐15a/16 cluster) (Zhang *et al*., 2012a), HDAC3 and EZH2 (miR‐26a, miR‐19, and miR‐129) (Han *et al*., 2016; Zhang *et al*., 2012b; Zhao *et al*., 2013), or DNMT3a (miR‐34a) (Craig *et al*., 2011). Repression of the members of the let‐7 family by MYC is regulated post‐transcriptionally by the MYC‐induced RNA binding protein Lin28B (Chang *et al*., 2009).

One of the first identified MYC‐regulated lncRNAs is CCAT1. While the CCAT1‐L transcript variant is specifically overexpressed in colorectal cancer, the CCAT1‐S variant is upregulated in many other cancers, including gastric carcinoma and colon cancer (He *et al*., 2014; Yang *et al*., 2013a). By binding to the E‐box element in the promoter region of *CCAT1*, MYC induces expression of CCAT1‐S. As the short transcript variant is most likely formed by 3′ processing of the long variant, MYC probably induces expression of CCAT1‐L, but this has not been proven. Besides CCAT1 and CCAT2, six other colorectal cancer‐associated MYC‐regulated lncRNAs (MYCLos/CCAT3‐8) have been identified (Kim *et al*., 2015a,b). Three of them are MYC‐induced, and the other three are MYC‐repressed. In the last five years, many more MYC‐regulated lncRNAs have been identified although for many their function has not yet been identified (Hart *et al*., 2014; Winkle *et al*., 2015).

**Figure 3:**
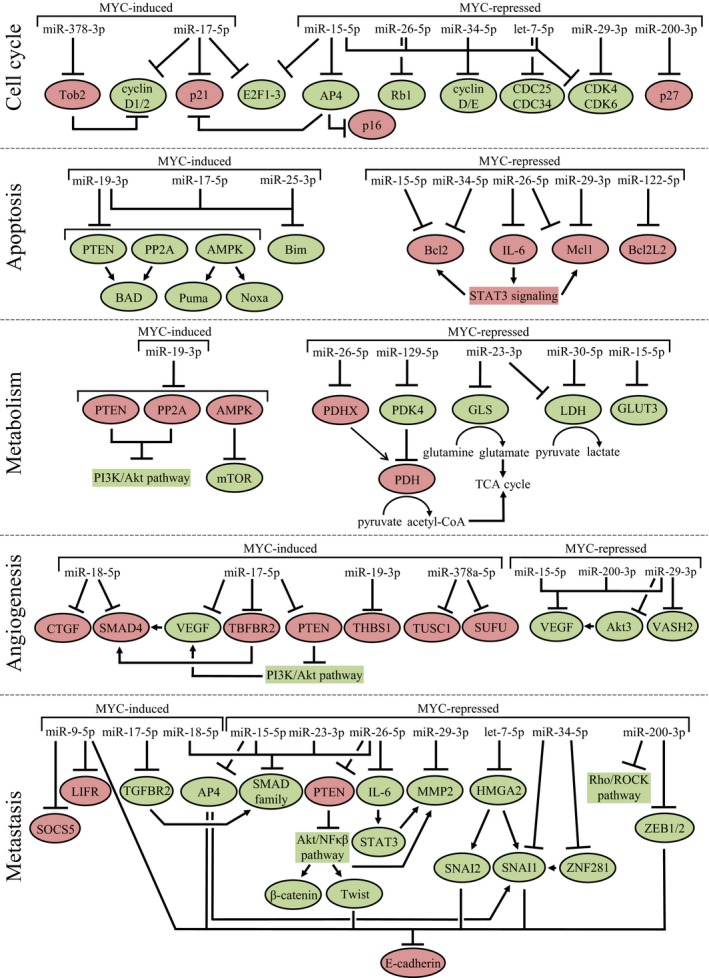
MYC‐regulated miRNAs involved in five important hallmarks of cancer. For each hallmark, the MYC‐regulated miRNAs and their protein targets involved in stimulation (green) or repression (red) of the respective hallmark are indicated.

**Figure 4:**
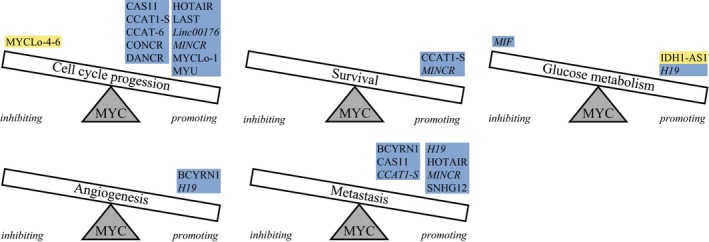
MYC‐regulated lncRNAs involved in five important hallmarks of cancer. For each hallmark, the lncRNAs that are promoting or inhibiting are indicated. Cell survival represents the opposite of apoptosis in this figure. LncRNAs highlighted in blue are MYC‐induced, lncRNAs highlighted in yellow are MYC‐repressed, and lncRNAs in italic function as sponges for miRNAs.

Below, we describe in more detail the MYC‐regulated miRNAs (Table 1 and Fig. 3) and lncRNAs (Table 2 and Fig. 4) with a clear role in five main hallmarks of cancer, that is, cell cycle progression, apoptosis, metabolism, angiogenesis, and metastasis.

**Table 1:** MYC‐regulated miRNAs with a function related to important hallmarks of cancer

	Proven target gene(s)^a^	Cellular processes^b^
MYC‐induced		
miR‐9‐5p	*CDH1, LIFR, SOCS5*	Angiogenesis, metastasis
miR‐17‐5p	*BIM, CCND1/2, E2F1‐3, CDKN1A, PTEN, TGFBR, VEGF*	Cell cycle progression, angiogenesis, apoptosis, metastasis
miR‐18‐5p	*CTGF, SMAD4*	Angiogenesis, metastasis
miR‐19‐3p	*AMPK, BIM, PP2A, PTEN, THBS1*	Apoptosis, angiogenesis, metabolism
miR‐25‐3p	*BIM, USP28*	Cell cycle progression
miR‐378‐3p	*TOB2*	Cell cycle progression
miR‐378a‐5p	*SUFU, TUSC1*	Angiogenesis
MYC‐repressed		
let‐7‐5p	*CCND2, CDC25, CDC34, CDK6, HMGA2*	Cell cycle progression, metastasis
miR‐15‐5p	*AP4, BCL2, CCND1/E1, CDK6, E2F3, GLUT3, SMAD3, VEGF*	Cell cycle progression, apoptosis, metabolism, angiogenesis
miR‐23‐3p	*GLS, LDHA/B, SMAD3‐5*	Metabolism, metastasis
miR‐26‐5p	*CCND2/E1‐2, CDK6, E2F3, EZH2, IL‐6, MCL1, PDHX, PTEN, RB1*	Cell cycle progression, apoptosis, metabolism, metastasis
miR‐29‐3p	*AKT3, CDK6, MCL1, MMPP2, VASH2, VEGF*	Cell cycle progression, apoptosis, angiogenesis, metastasis
miR‐30‐5p	*LDHA, UBE3C*	Metabolism, MYC regulation
miR‐34‐5p	*BCL2, CCND1/E2, CDK4, CDK6, SNAI1, ZNF281*	Cell cycle progression, apoptosis, metastasis
miR‐122‐5p	*BCL2L2*,* E2F1, TFDP2*	Apoptosis
miR‐129‐5p	*PDK4*	Metabolism
miR‐200‐3p	*AKT2, CDKN1B, CTNNB1, GIT2, ROCK2, VEGF, ZEB‐1, ZEB‐2*	Cell cycle progression, angiogenesis, metastasis

^a^Not all members of the seed families target the proven target genes. ^b^Not all target genes mentioned in column two are involved in the cellular processes mentioned here.

**Table 2:** MYC‐regulated lncRNAs with a function related to important hallmarks of cancer

	Proven target gene(s)	Cellular processes^a^
MYC‐induced		
BCYRN1	↑ *MMP2/9/13*,* VEGF*	Angiogenesis, metastasis
CASC11	↑ *HNRNPK*	Cell cycle progression
	↓ *WIF1*	Metastasis
CCAT1‐S	↑ BIRC7	Apoptosis
	↓ *CDKN1A*, let‐7a/b/c/e‐5p, miR‐148a‐3p	Cell cycle progression, metastasis
CCAT6	↓ *CDKN2B*	Cell cycle progression
CONCR	↑ *DDX11*	Cell cycle progression
DANCR	↓ *CDKN1A*, miR‐33b‐5p	Cell cycle progression
H19	↓ *CDH1*, let‐7a/b‐5p, miR‐29a‐3p, miR‐106a‐5p, miR‐200a‐c‐3p	Cell cycle progression, metabolism, angiogenesis, metastasis
HOTAIR	↓ *CDKN1A*,* WIF1*, miR‐34a‐5p	Cell cycle progression, metastasis,
LAST	↑ *CCND1*	Cell cycle progression
Linc00176	↓ miR‐9‐5p, miR‐185‐5p	Cell cycle progression
LncRNA‐MIF	↓ miR‐586‐5p	Metabolism
MINCR	↓ miR‐26a‐5p	Cell cycle progression, apoptosis, metastasis
MYCLo‐1	↓ *CDKN1A*	Cell cycle progression
MYU	↑ *CDK6*	Cell cycle progression
SINGH12	↑ *MMP13*	Metastasis
MYC‐repressed		
IDH1‐AS1	↑ *IDH1*	Metabolism
MYCLo‐4	↑ *GADD45A*	Cell cycle progression
MYCLo‐5	*Unknown*	Cell cycle progression
MYCLo‐6	↑ *GADD45A*	Cell cycle progression

↑ indicates induced/stabilized/activated by the lncRNA, and ↓ indicates being repressed by the lncRNA. ^a^Not all proven target genes mentioned in column two are involved in the cellular processes mentioned here.

### 3.1 Cell cycle progression

Nineteen MYC‐induced ncRNAs have a role in cell cycle progression. LncRNA‐assisted stabilization of transcripts (LAST) stimulates CCND1 expression by stabilizing CCND1 mRNA together with CNBP (Cao *et al*., 2017). MiR‐378a‐3p ensures CCND1 expression by targeting mRNA encoding TOB2, which is a repressor of CCND1 expression (Feng *et al*., 2011). CASC11 (CARLo‐7) promotes CCND1 transcription by stabilizing the hnRNP‐K mRNA, which leads to an hnRNP‐K‐dependent enhanced nuclear accumulation of β‐catenin (Zhang *et al*., 2016b). This leads to activation of WNT/β‐catenin signaling, and the subsequent induction of CCND1 transcription. The MYC‐induced lncRNA MY (VSP9D1‐AS1) associates with hnRNP‐K and stimulates CDK6 mRNA translation by competing with miR‐16‐5p for binding to CDK6 mRNA (Kawasaki *et al*., 2016). CDKN2B transcription is repressed by lncRNA CCAT‐6 upon binding of this lncRNA to hnRNP‐K (Kim *et al*., 2015b). All three lncRNAs interacting with hnRNP‐K (CASC11, MYU, and CCAT‐6) have been shown to stimulate cell cycle progression in colon cancer. The four lncRNAs HOTAIR, MYCLo‐1, CCAT1‐S, and DANCR all repress CDKN1A transcription (Kim *et al*., 2014, 2015b; Liu *et al*., 2013; Lu *et al*., 2018; Ma *et al*., 2014). HOTAIR represses CDKN1A transcription by recruiting EZH2 and inducing epigenetic changes, while MYCLo‐1 is assisted by HuR to repress the transcription of CDKN1A. The mechanisms by which CCAT1‐S and DANCR repress CDKN1A transcription are not yet known. Members of the miR‐17‐5p seed family have been strongly implicated in stimulation of cell cycle progression by targeting CDKN1A (Ivanovska *et al*., 2008; Kim *et al*., 2009; Trompeter *et al*., 2011). Conversely, the same seed family represses cell cycle progression by targeting CCND1/2 transcripts (Trompeter *et al*., 2011; Yu *et al*., 2008) and E2F1‐3 transcripts (He *et al*., 2005; Luan *et al*., 2018; O'Donnell *et al*., 2005; Trompeter *et al*., 2011). This is consistent with the cell type‐specific roles as oncomiR as well as tumor suppressor miR that have been observed for individual members of the miR‐17‐5p seed family (He *et al*., 2005; O'Donnell *et al*., 2005). The MYC‐induced lncRNA CONCR plays a role during S‐phase and is required for cell division by regulating the activity of helicase DDX11, which is involved in DNA replication and sister chromatid cohesion (Marchese *et al*., 2016). The MYC‐induced lncRNA MINCR promotes MYC‐mediated transcription of a selected set of cell cycle genes (Doose *et al*., 2015), although there is some debate about whether this lncRNA is a direct MYC‐induced lncRNA or not (Doose *et al*., 2015, 2016; Hart *et al*., 2016). Besides, MINCR functions as a sponge for miR‐26a‐5p to stimulate cell cycle progression (Wang *et al*., 2016).

Eleven MYC‐repressed ncRNAs inhibit cell cycle progression, while one MYC‐repressed miRNA stimulates cell cycle progression. The CCND1‐3 and CCNE1‐2 transcripts are targeted by let‐7b‐5p (Johnson *et al*., 2007), the miR‐15‐5p seed family (Bonci *et al*., 2008; Wang *et al*., 2009; Xu *et al*., 2009), miR‐26a/b‐5p (Kota *et al*., 2009; Zhu *et al*., 2012), and miR‐34a‐5p (He *et al*., 2007; Pok *et al*., 2013; Sun *et al*., 2008). In addition, these miRNAs and miR‐29a‐c‐3p target CDK4/6 transcripts (He *et al*., 2007; Johnson *et al*., 2007; Kawasaki *et al*., 2016; Sun *et al*., 2008; Xu *et al*., 2009; Zhao *et al*., 2010; Zhu *et al*., 2012). The RB1 transcript is targeted by miR‐26a‐5p (López‐Urrutia *et al*., 2017), and the E2F3 transcript is targeted by miR‐195‐5p, a member of the miR‐15‐5p seed family (Xu *et al*., 2009). Let‐7b‐5p targets the CDC25 transcript, which results in reactivation of CDKs to enable cell cycle progression (Hoffmann, 2000). Let‐7b‐5p also targets CDC34, which is an ubiquitin‐conjugating enzyme that is involved in the degradation of Wee1, an inhibitor of CDK1 (Legesse‐Miller *et al*., 2009). The miR‐15‐5p seed family members target TFAP4, which results in repression of CDKN1A and CDKN2A transcription and reduced p21 and p16 levels (Jackstadt *et al*., 2013a). MiR‐200b‐3p targets the CDKN1B transcript, leading to reduced p27 levels and stimulation of cell cycle progression (Fu *et al*., 2014). So, it seems not beneficial for cancer cells that MYC represses miR‐200b‐3p. MYCLo‐4 and MYCLo‐6 both block G2 to M phase progression by stimulating growth arrest and GADD45A expression, a critical regulator of G2 arrest (Kim *et al*., 2015a). MYCLo‐5 is involved in controlling S to G2 phase progression, but the exact mechanism is not yet known.

### 3.2 Apoptosis

Seven MYC‐induced and eight MYC‐repressed ncRNAs influence the balance between pro‐ and anti‐apoptotic factors. The MYC‐induced miR‐19a/b‐3p, miR‐20a‐5p, miR‐25‐3p, and miR‐92a‐3p prevent apoptosis by targeting the BIM transcript (Mogilyansky and Rigoutsos, 2013; Petrocca *et al*., 2008; Xiao *et al*., 2008). In addition, miR‐19a/b‐3p target transcripts of the PTEN, PP2A, and AMPK genes, resulting in decreased levels of the downstream pro‐apoptotic proteins BAD, Puma, and Noxa (Mavrakis *et al*., 2010; Mu *et al*., 2009; Olive *et al*., 2009). CCAT1‐S was shown to upregulate the expression of Livin, which is a member of the inhibitor of apoptosis protein family that can interact with caspases to prevent apoptosis (Chen *et al*., 2017).

Many of the MYC‐repressed miRNAs directly target anti‐apoptotic factors; for example, miR‐15a/16‐5p and miR‐34a‐5p target the BCL2 transcript (Bommer *et al*., 2007; Bonci *et al*., 2008; Cimmino *et al*., 2005), miR‐122‐5p targets the BCL2L2 transcript (Lin *et al*., 2008; Wang *et al*., 2014), and miR‐26b‐5p and miR‐29b‐3p target the MCL1 transcript (Jiang *et al*., 2015; Mott *et al*., 2007). Moreover, by targeting the IL‐6 transcript, miR‐26a‐5p represses STAT3 signaling, which results in reduced Bcl2 and Mcl1 expression levels (Yang *et al*., 2013b). The effects of miR‐26a‐5p can be antagonized by MYC‐induced MINCR, which functions as a sponge for this miRNA and prevent apoptosis (Wang *et al*., 2016).

### 3.3 Metabolism

Three MYC‐induced and eight MYC‐repressed ncRNAs are involved in the regulation of aerobic glycolysis, a feature of cancer cells. By targeting PTEN and PP2K transcripts, miR‐19a/b‐3p enhances PI3K activity (Mavrakis *et al*., 2010; Mu *et al*., 2009; Olive *et al*., 2009). This results in phosphorylation of Akt by PDK1, which stimulates glycolysis through multiple mechanisms, such as increased expression of several glucose transporters, activation of PFK1/2 (important regulatory enzymes of glycolysis), and mTOR. To further ensure high mTOR activity, miR‐19a/b‐3p also targets AMPK, an inhibitor of mTOR activity (Bolster *et al*., 2002; Mavrakis *et al*., 2010). MiR‐106a‐5p targets the E2F3 transcript, which results in repression of the glucose metabolism (Luan *et al*., 2018). This is antagonized by H19, which has been proposed to promote glucose metabolism by acting as a sponge for miR‐106a‐5p. MIF influences the glycolytic activity by sequestering miR‐586, thereby preventing expression of MYC target genes involved in glycolysis, that is, *GLUT1*,* LDHA*,* PKM2,* and *HK2* (Zhang *et al*., 2016a).

miRNAs repressed by MYC typically inhibit high metabolic activity. The initial uptake of glucose is regulated by miR‐195‐5p, which targets GLUT3 (Fei *et al*., 2012). MiR‐23a/b‐3p targets the mRNA encoding GLS, which converts glutamine to glutamate and thereby contributes to production of ATP (Gao *et al*., 2009). In addition, miR‐23a‐3p targets LDH subunits A and B (LDHA/LDHB), which convert the glycolytic end product pyruvate to lactate (Poyyakkara *et al*., 2018). Moreover, LDHA is also targeted by miR‐30a‐5p (Chang *et al*., 2008; Li *et al*., 2017a). MiR‐26a‐5p inhibits PDH activity by targeting PDHX and therefore inhibits the conversion of pyruvate to coenzyme A, an important component of the TCA cycle (Chen *et al*., 2014a). Instead, pyruvate is converted to lactate, showing an oncogenic role for miR‐26a‐5p in metabolism. In contrast, miR‐129 targets PDK4 mRNA, thereby stimulating PDH activity (Han *et al*., 2016). MYC‐repressed lncRNA IDH1‐AS1 stimulates homodimerization of IDH1 by forming a ternary structure with the enzyme, thereby enhancing its activity (Xiang *et al*., 2018). IDH1 converts isocitrate to α‐ketoglutarate, which is an intermediate in the TCA cycle and can inhibit glycolysis via degradation of HIF1α under normoxic condition (MacKenzie *et al*., 2007). By repressing IDH1‐AS1, MYC downregulates IDH1 activity and ensures glycolysis.

### 3.4 Angiogenesis

Stimulation of angiogenesis by different mechanisms has been reported for eight MYC‐induced ncRNAs, while five MYC‐induced and four MYC‐repressed miRNAs inhibit angiogenesis by targeting pro‐angiogenetic factors. Angiogenesis is enhanced by repression of the TGF‐β signaling pathway. MiR‐17‐5p and miR‐20a‐5p target the TGFBR2 transcript, while miR‐18a‐5p targets the downstream effector SMAD4 (Dews *et al*., 2010). Besides, several inhibitors of angiogenesis are targeted; miR‐19a‐3p targets THBS1 (Dews *et al*., 2010), miR‐18a‐5p targets CTGF (Ernst *et al*., 2010; Fox *et al*., 2013), and miR‐378‐5p targets TUSC2 and SUFU (Lee *et al*., 2007). VEGF expression is stimulated directly by lncRNA BCYRN1 (Hu and Lu, 2015; Peng *et al*., 2018) and indirectly by miR‐20a‐5p (Wang *et al*., 2017a). MiR‐20a‐5p targets PTEN, which leads to increased VEGF levels via activation of the PI3K/Akt pathway. In contrast, VEGF is inhibited by miR‐16‐5p, miR‐17‐5p, miR‐20a/b‐5p, miR‐29a‐3p, miR‐106a/b‐5p, and miR‐200b‐3p (Chen *et al*., 2014b; Choi *et al*., 2011; Hua *et al*., 2006). In this context, miR‐200b‐3p acts a tumor suppressor in contrast to its oncogenic role in cell cycle regulation. MiR‐29b‐5p indirectly lowers VEGF levels by targeting the Akt3 transcript (Li *et al*., 2017b). In melanoma cells, the effect of miR‐106a‐5p on VEGF expression can be counteracted by H19, which acts as a sponge for this miRNA (Luan *et al*., 2018). At first sight, it seems conflictive that both MYC‐induced and MYC‐repressed miRNAs target VEGF mRNA. However, as angiogenesis is crucial for a wide variety of physiological and pathological processes, VEGF expression has to be tightly regulated. This can be achieved by a combination of several regulatory factors including MYC‐induced and MYC‐repressed miRNAs, as well as other ncRNAs ensuring optimal VEGF levels under various conditions. MiR‐29a‐3p also targets the mRNA encoding a second pro‐angiogenetic factor, VASH2 (Jia *et al*., 2016). VASH2 inhibition by miR‐29a‐3p can also be antagonized by H19, which acts as a sponge for miR‐29a‐3p in glioma microvessels and epithelial cells (Jia *et al*., 2016).

### 3.5 Metastasis

Ten MYC‐induced ncRNAs target metastasis‐associated genes. H19 promotes metastasis by recruitment of EZH2 and the subsequent epigenetic suppression of E‐cadherin expression (Luo *et al*., 2013). Loss of E‐cadherin allows EMT, an early step in metastasis. MiR‐9‐5p promotes metastasis by targeting E‐cadherin, LIFR, and SOCS5 (Chen *et al*., 2012; Ma *et al*., 2010; Zhuang *et al*., 2012). LIFR inhibits metastasis through the Hippo/YAP pathway, and SOCS5 inhibits endothelial cell migration by inhibiting the JAK/STAT pathway. By interacting with EZH2, CASC11 and HOTAIR epigenetically suppress Wif1 expression and ensure stimulation of metastasis by the Wnt/β‐catenin pathway (Ge *et al*., 2013; Zhang *et al*., 2016b). As described in the paragraph above, three members of the miR‐17‐5p seed family target genes involved in the TGFβ signaling pathway, a crucial pathway also for the induction of metastasis. BCYRN1 stimulates metastasis by inducing the expression of MMP2, MMP9, and MMP13 (Hu and Lu, 2015; Peng *et al*., 2018). SNHG12 is a second lncRNA that induces the expression of MMP13 (Wang *et al*., 2017b). In contrast to BCYRN1 that induces MMP13 transcription, SNHG12 enhances MMP13 expression at the post‐transcriptional level.

Ten MYC‐repressed miRNAs prevent metastasis, while one MYC‐repressed miRNA can both induce and prevent metastasis, depending on the cell type. The transcription factors SNAI1/2, ZEB1/2, Twist, and AP4 all repress E‐cadherin expression at the transcriptional level (Tania *et al*., 2014). MiR‐34a‐5p targets the SNAI1 transcript directly and indirectly by targeting the Krüppel‐type transcription factor ZNF281 transcript (Hahn *et al*., 2013). In addition to being repressed by MYC, miR‐34a is also repressed by HOTAIR upon interaction with EZH2, thereby promoting metastasis in gastric cancer cells (Liu *et al*., 2015b). Let‐7a/b/e‐5p repress SNAI1 and SNAI2 expression indirectly by targeting the chromatin remodeling HMGA2 transcript (Lee and Dutta, 2007; Mayr *et al*., 2007). This is counteracted by CCAT1‐S functioning as a sponge for let‐7 family members let‐7a/b/c/e‐5p (Deng *et al*., 2015). CCAT1‐S can also sequester miR‐148a‐3p in osteosarcoma cells, thereby stimulating invasion and migration via unknown mechanisms (Zhao and Cheng, 2017). ZEB1/2 transcripts are targeted by miR‐200a‐c‐3p (Korpal *et al*., 2008; Park *et al*., 2008). The miR‐15a‐5p seed family targets mRNA encoding AP4, which induces SNAI1 expression (Jackstadt *et al*., 2013b). The role of miR‐26a‐5p with respect to metastasis seems to be contradictory. By targeting PTEN mRNA, miR‐26a‐5p stimulates the Akt/NFκB pathway and thereby induces expression of Twist, β‐catenin, and MMP2 in lung cancer (Liu *et al*., 2012). Increased levels of β‐catenin will initiate Wnt signaling, which stimulates metastasis. MMP2 is an essential protease involved in adhesion, invasion, and migration by proteolytic degradation of type IV collagen. In contrast, by targeting IL‐6 in hepatocellular carcinoma, miR‐26a‐5p represses STAT3 signaling and this results in lower MMP2 levels (Yang *et al*., 2013b). Furthermore, MMP2 is also targeted by miR‐29b‐3p (Fang *et al*., 2011). miRNAs that repress metastasis by repressing the downstream SMAD proteins of the TGFβ signaling pathway are miR‐23b‐3p (SMAD3‐5) (Rogler *et al*., 2009) and miR‐195‐5p (SMAD3) (Zhou *et al*., 2016). MiR‐200a‐3p targets the mRNA encoding β‐catenin in colorectal cancer, thereby repressing metastasis (Yang *et al*., 2017). Another pathway involved in metastasis by influencing cell motility is the Rho/ROCK signaling pathway, which is repressed by targeting of ROCK2 and GIT2 transcripts by miR‐200b/c‐3p (Peng *et al*., 2013; Wong *et al*., 2015; Zhou *et al*., 2017). All repressing effects of the miR‐200 seed family can be antagonized by H19, which functions as a sponge for these miRNAs (Li *et al*., 2016; Liang *et al*., 2015; Yang *et al*., 2017; Zhou *et al*., 2017). Besides, MINCR stimulates metastasis by sequestering miR‐26a‐5p (Wang *et al*., 2016).

## 4 Discussion

It is evident that an extensive, multilayered ncRNA network exists around MYC with critical roles for multiple lncRNAs and miRNAs in crucial cellular processes and in tumorigenesis. The picture that we present here is most likely still far from complete, as functions of most of the MYC‐regulated ncRNAs are not known yet (Hart *et al*., 2014; Robertus *et al*., 2010; Winkle *et al*., 2015). It is clear that many miRNAs and lncRNAs regulate MYC and that they can do this via diverse mechanisms at the level of transcription, translation, protein stability, and functionality. This suggests that redundancy is important to ensure optimal MYC levels and thereby cell viability under various conditions, as well as in different cell types. As MYC is involved in many cellular processes in redundant ways, it is remarkable that repression or reintroduction of a single MYC‐regulated ncRNA can already show strong effects on MYC‐associated phenotypes, as has been shown for many ncRNAs described in this review.

Expression of lncRNAs was shown to be more cell type specific than that of protein‐coding genes (Derrien *et al*., 2012). Also compared to miRNAs, lncRNAs appear to be more cell type‐specific. However, this might be biased as there are many more lncRNAs than miRNAs, which increases the chance to find cell type‐specific lncRNAs. Based on current knowledge, it seems that the cell type‐specific expression of certain lncRNAs can influence the output of MYC in two ways. First, cell type‐specific lncRNAs can influence important cellular processes downstream of MYC (Fig. 4). Second, other cell type‐specific lncRNAs, like PCGEM1 and PDIA3P, can modulate binding efficiency of MYC to promoters of a specific set of genes. So, these lncRNAs may direct the cell type‐specific target gene repertoire of MYC, rather than MYC acting as a general amplifier of expression. Altogether, a picture is emerging that lncRNAs guide cell type‐specific effects of MYC.

Although MYC has a central role in tumorigenesis, no effective MYC‐specific drugs are being employed in the clinic to date. Given the crucial functions of multiple lncRNAs and miRNAs in the oncogenic MYC network, it is tempting to speculate that targeting of ncRNAs within the MYC network might be an alternative to explore novel anticancer therapies. These ncRNAs can have profound impacts on MYC levels and activity and can also act downstream of MYC enabling cancer cells to gain the crucial hallmarks of cancer. To allow selection of the most optimal ncRNA targets, a more systematic analysis of their functional networks in normal cells as well as in cancer cells needs to be performed to oversee the consequences of targeting them.

Currently, more and more institutes and companies investigate how to specifically target miRNAs and lncRNAs, using both antisense and small molecule‐based strategies (Chakraborty *et al*., 2017; Warner *et al*., 2018). Inhibitors for miR‐92 and miR‐122, as well as mimics of miR‐16, miR‐29 and miR‐34, have been developed and tested or are currently tested in clinical trials (NIH U.S. National Library of Medicine, https://clinicaltrails.gov/ (accessed 06.08.2018)). As miR‐34a‐5p has tumor suppressor activity by both targeting MYC and stimulating apoptosis, while repressing cell cycle progression and metastasis, it is an attractive target for novel anticancer therapies. MiR‐16‐5p and miR‐29‐3p too have tumor‐suppressive roles in four of the five hallmarks discussed and form attractive targets as well. The cell type‐specific expression of lncRNAs adds to their attractiveness as targets for therapy (Derrien *et al*., 2012). The choice for an attractive target will therefore depend on the type of cancer. For example, CCAT1‐L and CCAT2 form attractive targets to specifically inhibit MYC transcription in colorectal cancer. A drug against CCAT1‐L, which will also target CCAT1‐S, would be very interesting as it will inhibit cell cycle progression and metastasis, while promoting apoptosis. However, a main problem for testing effectivity of lncRNA‐based drugs is the limited conservation for many of the lncRNAs, which prevents pre‐clinical experiments in relevant mouse models. Patient‐derived xenotransplantation models or organoid cultures might represent an alternative approach to test effectiveness of targeting human‐specific lncRNAs.

Thus, although MYC is described as one of the most important oncogenes, it is important to realize that there is an extensive, multilayered ncRNA network around MYC, in which intricate crosstalk contributes to hallmarks of cancer.

## Author contributions

LJYMS, AD‐K, AvdB, and JK conceived the outline, and LJYMS, AD‐K, MW, AvdB, and JK wrote the manuscript. LJYMS, AD‐K, and JK made the figures. LJYMS, AD‐K, MW, AvdB, and JK critically read the manuscript and LJYMS, AvdB, and JK finalized the manuscript.

## Conflicts of interest

The authors have no conflicts of interest to declare.
